# Summer Research Internship Curriculum to Promote Self-Efficacy, Researcher Identity, and Peer-to-Peer Learning: Retrospective Cohort Study

**DOI:** 10.2196/54167

**Published:** 2025-02-03

**Authors:** Yulia A Levites Strekalova, Rachel Liu-Galvin, Samuel Border, Sara Midence, Mishal Khan, Maya VanZanten, John Tomaszewski, Sanjay Jain, Pinaki Sarder

**Affiliations:** 1 Department of Health Services Research, Management and Policy, College of Public Health and Health Professions, University of Florida, Gainesville, FL, United States; 2 Ann & Robert H Lurie Children's Hospital of Chicago, Chicago, IL, United States; 3 Department of Pathology and Anatomical Sciences, University at Buffalo, Buffalo, NY, United States; 4 John T Milliken Department of Medicine, Washington University, St. Louis, MO, United States

**Keywords:** artificial intelligence, biomedical research, curriculum, training programs, workforce

## Abstract

**Background:**

Common barriers to students’ persistence in research include experiencing feelings of exclusion and a lack of belonging, difficulties developing a robust researcher identity, perceptions of racial and social stigma directed toward them, and perceived gaps in research skills, which are particularly pronounced among trainees from groups traditionally underrepresented in research. To address these known barriers, summer research programs have been shown to increase the participation and retention of undergraduate students in research. However, previous programs have focused predominantly on technical knowledge and skills, without integrating an academic enrichment curriculum that promotes professional development by improving students’ academic and research communication skills.

**Objective:**

This retrospective pre-then-post study aimed to evaluate changes in self-reported ratings of research abilities among a cohort of undergraduate students who participated in a summer research program.

**Methods:**

The Human BioMolecular Atlas Program (HuBMAP) piloted the implementation of a web-based academic enrichment curriculum for the Summer 2023 Research Internship cohort, which was comprised of students from groups underrepresented in biomedical artificial intelligence research. HuBMAP, a 400-member research consortium funded by the Common Fund at the National Institutes of Health, offered a 10-week summer research internship that included an academic enrichment curriculum delivered synchronously via the web to all students across multiple sites. The curriculum is intended to support intern self-efficacy, researcher identity development, and peer-to-peer learning. At the end of the internship, students were invited to participate in a web-based survey in which they were asked to rate their academic and research abilities before the internship and as a result of the internship using a modified Entering Research Learning Assessment instrument. Wilcoxon matched-pairs signed rank test was performed to assess the difference in the mean scores per respondent before and after participating in the internship.

**Results:**

A total of 14 of the 22 undergraduate students who participated in the internship responded to the survey. The results of the retrospective pre-then-post survey indicated that there was a significant increase in students’ self-rated research abilities, evidenced by a significant improvement in the mean scores of the respondents when comparing reported skills self-assessment before and after the internship (improvement: median 1.09, IQR 0.88-1.65; W=52.5, *P*<.001). After participating in the HuBMAP web-based academic enrichment curriculum, students’ self-reported research abilities, including their confidence, their communication and collaboration skills, their self-efficacy in research, and their abilities to set research career goals, increased.

**Conclusions:**

Summer internship programs can incorporate an academic enrichment curriculum with small-group peer learning in addition to a laboratory-based experience to facilitate increased student engagement, self-efficacy, and a sense of belonging in the research community. Future research should investigate the impact of academic enrichment curricula and peer mentoring on the long-term retention of students in biomedical research careers, particularly retention of students underrepresented in biomedical fields.

## Introduction

Several population groups continue to be underrepresented among trainees pursuing biomedical research disciplines [1-5]. According to 2023 census data, Hispanic, Black, and American Indian and Alaska Native individuals comprised 34.5% of the US population (19.5%, 13.7%, and 1.3%, respectively) [6]. Yet, only 16.0% of students awarded science and engineering doctorate degrees in 2020 were from these groups [7]. These disparities carry over into the workforce, with Hispanic individuals comprising only 15.0% of the STEM (science, technology, engineering, and math) workforce in 2021, 9.0% of Black individuals, and <1.0% of American Indians and Alaska Native individuals [7]. These figures illustrate a disparity between the racial and ethnic diversity of the US population and of individuals working in biomedical and STEM fields.

The underrepresentation of trainees from diverse backgrounds is particularly pronounced in the fields of biomedical artificial intelligence (AI) and machine learning [8,9]. Biomedical research has seen significant growth in AI applications with the development of tools for diagnosing, monitoring, and predicting prognoses [10,11]. With AI’s increasing role in society, AI researchers’ diversity must mirror the diverse populations they serve. A skilled research workforce in domain-specific AI applications is needed to maintain sustained progress in AI research. According to a 2019 report from the AI Now Institute, women comprised only 18% of authors at AI conferences, while 80% of AI professors were male [8]. A lack of diverse perspectives can have significant consequences on resulting research products, as lack of fairness (the ability of AI to be free from bias) and explainability (the ability to understand why an AI model makes certain predictions, which is crucial for identifying biases) have plagued the AI field for years [10,11].

Common barriers to students’ participation and persistence in research include experiencing feelings of exclusion and a lack of belonging, difficulties developing a strong research identity, perceptions of racial and social stigma directed toward them, and perceived gaps in research skills [1-3,12,13]. One study, for example, observed that reasons for dropout from computer science degrees included unclear expectations about the chosen field, feeling as if they did not belong, and social isolation [12]. These challenges are particularly pronounced among trainees from groups traditionally underrepresented in research, as articulated by the National Institutes of Health (NIH) [14]. The NIH reports that several racial and ethnical groups are underrepresented in research: Black or African American individuals, Hispanic or Latinx individuals, American Indians or Alaska Natives, and Native Hawaiians and other Pacific Islanders, with women carrying a higher burden of underrepresentation [14]. To address these known barriers, research exposure programs for undergraduate students can include a curriculum of academic skill development components that promote student self-efficacy, identity, and a sense of belonging in research. Specifically, evidence shows that students encouraged to develop their “science identity” are more likely to integrate into the wider culture of scientific research [3]. Students can develop their self-confidence, self-efficacy, and identity as scientists through science support experiences and connections with peers and mentors, thus benefiting from exposure to diverse perspectives and experiences [5,15,16]. Additionally, experiences that foster a sense of belonging to the research community have been shown to lead to greater persistence in STEM fields, especially for students from groups underrepresented in research [16-19]. Furthermore, STEM undergraduates who engage in small-group and active learning demonstrate increased self-efficacy, achieve better grades, and are more likely to remain in STEM fields [16,20,21].

There is a critical need for nationwide collaborative training programs to fund, develop, and mentor students from underrepresented groups to ensure AI representation in biomedical research [22]. Summer research programs delivered via both in-person and web-based formats have been shown to increase the participation and retention of undergraduate students in research [23]. Evidence shows that involvement in both face-to-face and remote summer research experiences equips undergraduate students with knowledge and research skills and promotes enrollment in postgraduate training and entry into scientific- or health care–related careers [24,25]. In addition to being exposed to research and learning research skills, students can also gain confidence as scientists and develop a researcher identity and intention to pursue a career in STEM [19,26,27]. However, previous programs have primarily focused on equipping students with technical knowledge and skills, with varying degrees of emphasis on professional development, without integrating a universal academic enrichment curriculum that is delivered synchronously via the web to students across multiple sites and that focuses on active learning and improving students’ academic and research skills [23-27]. The effective development of students’ research skills and familiarity with scientific methods calls for supplementing laboratory research experience with academic enrichment sessions in order to equip students with the necessary academic and professional skills to thrive as researchers building their identities in the research space. These sessions should allow students to actively engage in discussions about their identities as researchers while offering a supportive environment that fosters a sense of belonging and facilitates peer-to-peer information exchange. This study aimed to report the findings of delivering academic enrichment sessions to undergraduate students from groups underrepresented in AI research and to test the hypothesis that a guided academic enrichment curriculum, as part of a summer research internship that also included a laboratory-based experience, promotes self-perceived research skills, researcher identity, and professional skills.

## Methods

### Program Background

The Human BioMolecular Atlas Program (HuBMAP) is a consortium of diverse research teams with expertise in biology, imaging, computation, data management, and visualization. The program is funded by the NIH Common Fund and aims to create a multiscale spatial atlas of the human body at single-cell resolution by applying advanced technologies and disseminating resources to the community [28]. Extensive infrastructure has been established through a coordinated effort among various HuBMAP integration, visualization, and engagement teams; tissue-mapping centers; technology and tools development groups; and rapid technology implementation teams and working groups [29]. To ensure the pipeline of emerging research talent familiar with HuBMAP’s tools, data, and processes, the program began offering summer research internships for undergraduate students in 2021. The internship included placement of the student within an active research laboratory for 8‐10 weeks, during which time they were mentored by an established investigator, and at the end of which they were encouraged to deliver a presentation of their research project. The 2023 HuBMAP internship program hosted students at 19 different research laboratories across 12 different institutions. Participants were recruited nationally by the NIH coordinating center; they submitted applications that included a research interest and career goal statement and were selected for a paid internship based on matching their research interests to those of participating mentors. A complete list of participating laboratories and institutions is included in Multimedia Appendix 1.

In 2023, the HuBMAP internship program was enhanced with an academic enrichment curriculum. The curriculum was informed by the evidence-based Entering Research curriculum, developed by a faculty trained as an Entering Research facilitator [30], and designed to address the needs and challenges of a diverse group of undergraduate students from groups underrepresented in biomedical and AI research. The academic enrichment curriculum included a series of interactive workshops, peer discussions, and hands-on writing assignments aimed at supporting students to develop their research identities and enhance their research communication skills. Each session lasted 2 hours and was delivered synchronously via Zoom, enabling students participating in internships at 19 different research laboratories across 12 different institutions across the United States to attend. A complete list of the sessions constituting the academic enrichment curriculum can be found in Multimedia Appendix 2. Sessions were structured into 1 hour of didactics, small-group activities, and discussions and 1 hour of guided work on academic products. Examples of academic products included a research statement, an individual development plan, a web-based ORCID profile, and a research poster, among others. An institutional Google Drive account was used to capture interns’ work systematically and securely, promote peer-to-peer learning, and facilitate continuous access to training materials and assignments.

### Data Collection and Instruments

In the summer of 2023, a total of 22 students participated in the HuBMAP summer internship, and their self-reported demographic data were collected through the initial application form. Demographic variables collected during the application process included sex, race and ethnicity, class standing, degree major, and home institution type (categorized as per Carnegie classification).

At the end of the internship, all 22 students were invited to participate in a web-based survey in which they were asked to rate their research skills self-efficacy (operationalized on the survey instrument as academic and research abilities) before the internship (referred to as the before questions) and as a result of the internship (referred to as the after questions). It is important to note that the after questions specifically asked the students to rate their abilities as a result of the internship, and not as a result of the academic enrichment curriculum. The survey employed a 17-item scale and validated questions adapted from the Entering Research Learning Assessment instrument [31]. The Entering Research Learning Assessment instrument is designed to measure learning gains of undergraduate or graduate research trainees across 7 areas: research comprehension and communication skills, practical research skills, research ethics, researcher identity, equity and inclusion awareness and skills, and professional and career development skills. The survey was piloted with undergraduate students and presented at several meetings to internship leaders at the NIH and participating sites to gain expert feedback and ensure the content and external validity of the items. Examples of questions in the survey included those that asked about students’ self-reported ability to communicate the context, methods, and results of their research, to tailor research communications to different audiences, to work comfortably in the research environment, to explore possible research career pathways, to set research career goals, and to develop a plan to pursue a research career. For each item, students were asked to indicate their abilities on a scale of 1‐5, with 1 representing “no ability,” 2 representing “a little ability,” 3 representing “moderate ability,” 4 representing “good ability,” and 5 representing “great ability.” The complete list of survey questions can be found in (Multimedia Appendix 3).

The retrospective pre-then-post survey was administered anonymously, and the participants were asked to rereport their sex, race and ethnicity, and class standing. Since the retrospective pre-post survey was anonymous, we were unable to tell which students had or had not responded to the survey; thus, we compared the demographic characteristics of the respondents (n=14) to that of the whole cohort (N=22).

### Statistical Analyses

The Cronbach α was applied to measure the internal consistency of the items included to assess the participants’ research skills and self-efficacy. Median, minimum, and maximum scores for each of the before and after questions were calculated. Mean scores per respondent for the before questions compared with the after questions were also calculated, and a Wilcoxon matched-pairs signed rank test was performed to assess the median difference in the mean scores per respondent for the before questions compared with the after questions. Although the survey results were anonymous, we were able to match responses to each before and after question to individual survey respondents based on anonymous identification numbers.

All statistical analyses were performed using SAS (version 9.4; SAS Institute Inc). All *P* values were 2-sided, with *P*<.05 considered statistically significant.

### Ethical Considerations

The study was submitted for review to the University of Florida Institutional Review Board and determined to be exempt (#ET00041828). Informed consent was obtained from all survey respondents. No compensation was provided for participants in this study, and no identification of individual participants in any manuscript or supplementary material is possible.

## Results

### Participant Characteristics

Participants in the 2023 HuBMAP summer internship and curriculum included 22 students from undergraduate institutions across the United States. Students had declared majors in STEM fields such as biology, bioinformatics, biochemistry, and engineering. While class standing varied among the participants, half of the students were at a sophomore level before beginning their summer internship. Related to their incoming career goals, participants often reported interest in pursuing MD, PhD, or MD/PhD degrees and sought to use the internship to explore the various career opportunities available in biomedicine. Almost half of the participants (9/22, 40.9%) were from high (R2) and very high (R1) research activity academic institutions, with the rest of the participants (13/22, 59.1%) from master, baccalaureate, and associate colleges. Demographic information of the 22 students is presented in Table 1.

**Table 1.** Characteristics of Human BioMolecular Atlas Program summer 2023 interns (N=22) and survey respondents (N=14).

Demographic	Interns, n (%)	Survey respondents, n (%)
Sex		
Male	7 (31.8)	3 (21.4)
Female	14 (63.6)	10 (71.4)
Other	1 (4.5)	1 (7.1)
Race		
Black or African American	6 (27.3)	5 (35.7)
Asian or Pacific Islander	5 (22.7)	3 (21.4)
White	4 (18.2)	1 (7.1)
Other race	0 (0)	1 (7.1)
More than one race	2 (9.1)	1 (7.1)
N/A^a^ or prefer not to answer	5 (22.7)	3 (21.4)
Ethnicity		
Hispanic or Latino	7 (31.8)	3 (21.4)
Not Hispanic or Latino	15 (68.2)	11 (78.6)
Class standing		
Freshman	5 (22.7)	2 (14.3)
Sophomore	11 (50.0)	7 (50.0)
Junior	3 (13.6)	2 (14.3)
Senior	3 (13.6)	3 (21.4)
Major		
Biology	8 (36.4)	—^b^
Biochemistry	5 (22.7)	—
Bioinformatics	2 (9.1)	—
Chemistry	1 (4.5)	—
Biomedical/electrical engineering	2 (9.1)	—
Genetics	1 (4.5)	—
Information technology	1 (4.5)	—
Nanomedicine	1 (4.5)	—
Neuroscience	1 (4.5)	—
Home institution type per Carnegie classification		
Doctoral universities: very high research activity (R1)	5 (22.7)	—
Doctoral universities: high research activity (R2)	4 (18.2)	—
Master colleges and universities: larger programs (M1)	4 (18.2)	—
Master colleges and universities: smaller programs (M3)	1 (4.5)	—
Baccalaureate colleges	7 (31.8)	—
Associate colleges	1 (4.5)	—

a N/A: not applicable.

b Not available.

Invitations to complete a web-based survey were sent out to all 22 students, and 14 survey responses were received and included in data analysis. The makeup of the survey respondents aligned with the overall sample, but the sample size was too small for statistical analyses. The demographic makeup of all interns who participated in the program and the subset who responded to the survey are reported in Table 1. The postprogram survey did not collect data on degree major or institutional affiliation; thus, this information is not reported for the Survey Respondents column in Table 1.

### Results of Retrospective Pre-Post Survey

Cronbach α was calculated to measure the level of internal consistency among the survey questions, and its value of 0.96 for the preintervention questions and 0.92 for the postintervention questions indicated an excellent level of internal consistency, that is, all questions in the survey were assessing the primary underlying concepts being assessed: researcher skills, researcher identity, and self-efficacy in research.

Table 2 reports the median, minimum, and maximum values of the responses to each of the before questions compared with each of the after questions. Given the ordinal nature of the data, where the median was a decimal number due to the averaging of 2 middle values, the median was rounded up to the nearest whole number. Of all survey responses, 3 responses were missing (one to each of After Question 3, After Question 5, and After Question 9); missing responses therefore comprised <1% of all data.

**Table 2.** Median and range of responses to self-reported research skills assessment before versus after questions.

Skill assessed	Before, median (IQR; min, max)	After, median (IQR; min, max)
Research comprehension and communication skills		
Communicate the context, methods, and results of your research.	3 (2-4.25; 1, 5)	4 (4-5; 3, 5)
Tailor your research communications for different audiences (eg, general public and disciplinary conference)	3 (3-4; 1, 5)	5 (4-5; 4, 5)
Work in the research environment comfortably	3 (2-4.25; 1, 5)	5 (4-5; 4, 5)
Ask questions to clarify your understanding of your research project	4 (3-4; 1, 5)	5 (4-5; 4, 5)
Practice regular and open communication with your research mentor	3 (2.75-4; 1, 5)	5 (4-5; 4, 5)
Practice regular and open communication with your research team members	3 (2.75-4; 1, 5)	5 (4-5; 4, 5)
Practical research skills		
Use the tools, materials, and equipment needed to conduct research	3 (2-3.25; 1, 5)	5 (4-5; 4, 5)
Make a case for your research question based on the literature	3 (2-3; 1, 5)	4 (4-5; 3, 5)
Research ethics		
Identify forms of unethical practices or research misconduct	3 (2.75-4; 1, 5)	4 (4-5; 3, 5)
Researcher identity		
Think of yourself as a scientist/researcher	3 (2-3; 1, 5)	4 (3.75-5; 2, 5)
Feel like you belong in research	3 (2-3.25; 1, 5)	4 (3-5; 2, 5)
Researcher confidence and independence		
Work independently on your research project	2 (1.75-3.25; 1, 4)	5 (4-5; 2, 5)
Determine the next steps in your research project	2 (1.75-3; 1, 5)	4 (3.75-5; 3, 5)
Equity and inclusion awareness and skills		
Understand how others might experience research differently based on their identity (eg, race, socioeconomic status, first-generation status)	3 (1-4; 1, 5)	5 (4-5; 1, 5)
Professional and career development skills		
Explore possible research career pathways	3 (2-4; 1, 5)	5 (3.75-5; 3, 5)
Set research career goals	3 (2-3; 1, 5)	5 (4-5; 3, 5)
Develop a plan to pursue a research career (determine the next step in your training)	3 (2-3.25; 1, 4)	5 (4-5; 3, 5)

The results from the Wilcoxon signed rank matched-pairs test indicated that there was a significant improvement in the mean scores of the respondents when comparing the before questions to the after questions (improvement: median 1.09, IQR 0.88-1.65; W=52.5, *P*<.001). The mean scores of each respondent for the before questions compared with the after questions are presented in Multimedia Appendix 4. A review of individual items revealed an increase in all items, indicating that overall, students felt their research abilities had improved following the HuBMAP Summer Research Internship.

## Discussion

This paper reports on the pilot implementation of an academic enrichment curriculum delivered as part of the summer, AI-focused, undergraduate research internship administered through the HuBMAP program. Following participation in the internship, students reported increases in self-assessed research skills, including their abilities to set research career goals, to think of themselves as researchers, and to work comfortably in the research environment, and increases in their feelings of belonging in research. Our curricular intervention included academic enrichment content supplemented by peer learning and hands-on activities.

Earlier research has demonstrated that perceived gaps in research skills and feelings of isolation have detrimental effects on students’ persistence in computer science and AI-focused training [12]. While most summer research internships focus on individual training and technical aspects of research with varying degrees of emphasis on professional development [23-27], the format and content of the supplemental academic enrichment curriculum we piloted showed promise in creating a nurturing, active, and collaborative learning environment for student interns across multiple institutions, with demonstrated self-reported improvements in abilities that went beyond technical research skills.

Based on our study’s findings, we have formulated propositions and hypotheses about the mechanisms through which academic enrichment curricula promote research skills self-efficacy and researcher identity and result in sustained engagement in research training. First, delivering an academic enrichment curriculum enhances and amplifies the learning experiences of students participating in summer research programs and promotes the long-term retention of these students in biomedical research careers. Second, academic enrichment sessions help to build a research community and promote a sense of belonging among students. Third, providing students with opportunities to learn from one another encourages peer learning and enhances students’ critical thinking skills, resourcefulness, and resilience within academia. Additional research questions that we would like to answer through future studies and longitudinal assessments include how students can learn from one another and whether peer learning is superior to learning from mentors. Future studies should also explore the effect of individual versus cohort-based research experiences. The academic enrichment curriculum we delivered to participants of the HuBMAP summer internship program would be considered a mix of cohort (delivered through the academic enrichment sessions) and hybrid (since students were based at separate laboratories and institutions with individual mentors) research experiences. In addition, further research is needed to determine the optimal structure of the academic enrichment curriculum, including the frequency of sessions (once weekly vs every other week, for example).

Our experience suggests that the integration of an academic enrichment curriculum into a laboratory-based AI-focused summer research internship can help students increase their confidence, communication skills, self-efficacy, and sense of belonging in the research community. In line with our findings, a growing body of evidence shows that a structured curriculum supports undergraduate students from traditionally underrepresented groups in developing their identity as scientists, and in their overall self-efficacy to use scientific thinking, through science-support experiences and connections formed with peers [5,15,16]. Thus, we recommend that future programs should incorporate small group peer learning, active learning strategies, and laboratory-based research experience as evidence-supported methods to facilitate increased self-efficacy and a sense of belonging in the research community, which may also have the potential to promote the retention of undergraduate students in STEM and biomedical disciplines [16,20,21].

Despite these encouraging results, it is important to recognize that the generalizability of the results of this study is limited by its relatively small sample size of 22 students, of which 14 students (63.6%) completed the pre- and postprogram surveys. As a result, this study’s findings may not fully represent the broader experiences of other students across other programs. It is also important to note that it was not possible to separate the effect of our academic enrichment curriculum from other activities that students may have been engaged in during their HuBMAP Summer Research Internship. Multiple individual laboratories across different institutions hosted students participating in the 2023 HuBMAP summer internship program. Therefore, while we delivered this academic enrichment curriculum to all students via the web, some students may have been provided additional learning opportunities (remote or in-person) at the laboratories or institutions where they were based. The nature and quantity of such other learning opportunities may have varied across the different sites and should be explored in future studies. It is possible that a portion of the gains in students’ self-reported research abilities were attributable to these supplementary activities and not because of the academic enrichment curriculum. Future research should therefore investigate the specific impact of academic enrichment curricula on the long-term retention of students in biomedical research careers, particularly retention of students underrepresented in biomedical fields. Furthermore, each student received one-to-one and group mentoring from lead investigators and senior trainees of the host labs. The effects of mentoring and the role of peer-to-peer interaction as contributing factors in student academic skill development can also be quantified and measured in future evaluations of summer research experiences. Additionally, this study used a pre-then-post evaluation design, which combined the questions in which students were asked to rate their research abilities “before” and “as a result of” the HuBMAP Summer Research Internship into a single survey administered post program. This methodological design allowed the students to assess knowledge gained from a perspective informed by a 10-week experience. However, the effects of recall and social desirability biases can be evaluated with sufficiently powered studies that use different evaluation formats.

Following their participation in the HuBMAP 2023 summer internship program, which included an academic enrichment curriculum delivered remotely, students’ self-reported research abilities, including their confidence and communication skills, their self-efficacy in research, and their abilities to set research career goals, increased. Students also reported an increased ability to think of themselves as researchers and to work comfortably in the research environment, as well as increased feelings of belonging in research after participating in the program. We recommend that summer internship programs incorporate an academic enrichment curriculum with small-group peer learning in addition to a laboratory-based experience to facilitate increased student engagement, self-efficacy, and a sense of belonging in the research community. Strengthening the pipeline of skilled and diverse professionals entering these fields will help in addressing both current and future workforce needs, reducing bias in AI fields, and cultivating the next generation of scientists and leaders in public health, with the goal of improving health outcomes for all populations.

## Supplementary material

10.2196/54167 Multimedia Appendix 1 List of research laboratories/institutions that hosted students for the 2023 Human BioMolecular Atlas Program summer internship program.

10.2196/54167 Multimedia Appendix 2 Schedule and objectives of academic enrichment sessions offered to summer 2023 Human BioMolecular Atlas Program interns.

10.2196/54167 Multimedia Appendix 3 Academic enrichment evaluation instrument.

10.2196/54167 Multimedia Appendix 4 Mean scores per respondent for before questions compared to after questions.

## Abbreviations

AI: artificial intelligence
HuBMAP: Human BioMolecular Atlas Program
NIH: National Institutes of Health
STEM: science, technology, engineering, and math
