# Supplementary material for: Summer Research Internship Curriculum to Promote Self-Efficacy, Researcher Identity, and Peer-to-Peer Learning: Retrospective Cohort Study
Source: JMIR Form Res. 2025 Feb 3;9:e54167. doi: 10.2196/54167 (PMC11809269; doi:10.2196/54167)
Supplement: Multimedia Appendix 1 [file formative-v9-e54167-s001.docx]

Multimedia Appendix 1. List of Research Laboratories/Institutions that Hosted Students for the 2023 HuBMAP Summer Internship Program.

| **Research Laboratory** | **Institution** |
| --- | --- |
| Anderton Lab | Pacific Northwest National Laboratory |
| Angelo Lab | Stanford University |
| Bendall Lab | Stanford University |
| Börner Lab | Indiana University |
| Fan Lab | Yale University |
| Gehlenborg Lab | Harvard Medical School |
| Hagood Lab | University of Rochester Medical Center |
| Jain Lab | Washington University in St. Louis School of Medicine |
| Kim, O’Neill, and Gregory Labs | University of Pennsylvania |
| Laurent Lab | University of California, San Diego |
| Pasa-Tolic Lab | Pacific Northwest National Laboratory |
| Pei Lab | University of Pennsylvania |
| Pryhuber Lab | University of Rochester Medical Center |
| Sarder Lab | University of Florida |
| Satija Lab | New York Genome Center |
| Segré Lab | Harvard University |
| Shi Lab | University of California, San Diego |
| Spraggins Lab | Vanderbilt University |
| Tan Lab | University of Pennsylvania |
