# Supplementary material for: Summer Research Internship Curriculum to Promote Self-Efficacy, Researcher Identity, and Peer-to-Peer Learning: Retrospective Cohort Study
Source: JMIR Form Res. 2025 Feb 3;9:e54167. doi: 10.2196/54167 (PMC11809269; doi:10.2196/54167)
Supplement: Multimedia Appendix 2 [file formative-v9-e54167-s002.docx]

Multimedia Appendix 2. Schedule and objectives of Academic Enrichment sessions offered to summer 2023 HuBMAP interns

| **Session*** | **Title** | **Learning Objectives** | **Portfolio Focus** |  |
| --- | --- | --- | --- | --- |
| 1 | Roles of Mentees and Mentors | 1. Formulate the expectations of their research mentoring relationship.  2. Develop a plan to guide career and professional development.  3. Establish a plan for communication with their mentor to address any differences and align their expectations. | Individual Development Plan |  |
| 2 | Defining Your Research Project | 1. Learn to engage general and expert audiences in a conversation about their research.  2. Practice explaining and asking questions about biomedical research. | Elevator Sentences |  |
| 3 | Ethics in Research | 1. Identify potential risks to the responsible conduct of research.  2. Develop strategies to deal with difficult situations that may arise during their research experiences. | Research Statement |  |
| 4 | Researcher Identity and Persistence | 1. Articulate personal strengths and reflect on research experiences.  2. Draft a research identity statement.  3. Review peers’ research identity statements. | Online Profile and ORCID ID |  |
| 5 | Grounding Your Research Project: Practical Reading and Notetaking Strategies for Research Papers | 1. Identify approaches to communicate research results to the public and academic community  2. Learn how to systematically read, understand, and evaluate a research paper using guided questions and critical thinking.  3. Learn about electronic tools for organizing papers, citing papers, and taking notes on papers.  4. Develop an annotated bibliography. | Annotated Bibliography |  |
| 6 | Interdisciplinary Collaborations & Team Science | 1. Understand the benefits of and be able to apply team science as a collaboration approach.  2. Understand the concepts of groups vs. teams, teaming, team-building, and team management.  3. Identify one's assets (such as skills, networks and connections, time, space, access to data, preliminary data, personnel, and technology) as well as the assets of others.  4. Design and discuss collaborative opportunities with another HuBMAP intern. | Research Project Summary |  |
| 7 | Communicating Research through Research Abstracts and Posters | 1. Understand the purpose of and be able to write a research abstract for your poster.  2. Learn about the key elements of a research poster, including figures and tables, and assemble a research poster.  3. Be familiar with the logistics of poster presentations, including how to present your poster findings and communicate professionally with readers. | Research Abstract and Poster |  |
| 8 | Graduate School 101 | 1. Identify the characteristics and attributes desired in a letter of recommendation.  2. Learn how to request a letter of recommendation from a mentor. | Letters of Recommendation |  |

* An additional planned session, "Exploring Research Careers: Career Panel Resume and Research,” was canceled due to a scheduling conflict.
