# Supplementary material for: Summer Research Internship Curriculum to Promote Self-Efficacy, Researcher Identity, and Peer-to-Peer Learning: Retrospective Cohort Study
Source: JMIR Form Res. 2025 Feb 3;9:e54167. doi: 10.2196/54167 (PMC11809269; doi:10.2196/54167)
Supplement: Multimedia Appendix 3 [file formative-v9-e54167-s003.docx]

Multimedia Appendix 3. Academic Enrichment Evaluation Instrument

*Block 1. Demographics*

Please tell us a bit about yourself.

**What is the highest level of academic training that you have completed?**

Freshman year

Sophomore year

Junior year

Senior year

Other

**What is your gender identity?**

Man

Woman

Transgender

Other

**What best describes your race and ethnicity? (select all that apply)**

African American/Black

Asian/Pacific Islander

Caribbean descent

Hispanic/Latino/a and/or of Spanish origin

Multiracial

Native American/American Indian

White

Other

*Block 2. Program Outcomes*

**BEFORE your HuBMAP Summer Research Internship, indicate your ability to …**

1. Communicate the context, methods, and results of your research. [Research Comprehension and Communication Skills]
2. Tailor your research communications for different audiences (e.g., general public, disciplinary conference). [Research Comprehension and Communication Skills]
3. Work in the research environment comfortably. [Research Comprehension and Communication Skills]
4. Ask questions to clarify your understanding of your research project. [Research Comprehension and Communication Skills]
5. Practice regular and open communication with your research mentor. [Research Comprehension and Communication Skills]
6. Practice regular and open communication with your research team members. [Research Comprehension and Communication Skills]
7. Use the tools, materials, and equipment needed to conduct research. [Practical Research Skills]
8. Make a case for your research question based on the literature. [Practical Research Skills]
9. Identify forms of unethical practices or research misconduct. [Research Ethics]
10. Think of yourself as a scientist/researcher. [Researcher Identity]
11. Feel like you belong in research. [Researcher Identity]
12. Work independently on your research project. [Researcher Confidence and Independence]
13. Determine the next steps in your research project. [Researcher Confidence and Independence]
14. Understand how others might experience research differently based on their identity (e.g., race, socioeconomic status, first-generation status). [Equity and Inclusion Awareness and Skills]
15. Explore possible research career pathways. [Professional and Career Development Skills]
16. Set research career goals. [Professional and Career Development Skills]
17. Develop a plan to pursue a research career (determine the next step in your training). [Professional and Career Development Skills]

All items on a scale

1 = “no ability”

2 = “a little ability”

3 = “moderate ability”

4 = “good ability”

5 = “great ability”

**AS A RESULT of your HuBMAP Summer Research Internship, indicate how much you gained in your ability to …**

1. Communicate the context, methods, and results of your research.
2. Tailor your research communications for different audiences (e.g., general public, disciplinary conference).
3. Work in the research environment comfortably.
4. Ask questions to clarify your understanding of your research project.
5. Practice regular and open communication with your research mentor.
6. Practice regular and open communication with your research team members.
7. Use the tools, materials, and equipment needed to conduct research.
8. Make a case for your research question based on the literature.
9. Identify forms of unethical practices or research misconduct.
10. Think of yourself as a scientist/researcher.
11. Feel like you belong in research.
12. Work independently on your research project.
13. Determine the next steps in your research project.
14. Understand how others might experience research differently based on their identity (e.g., race, socioeconomic status, first-generation status).
15. Explore possible research career pathways.
16. Set research career goals.
17. Develop a plan to pursue a research career (determine the next step in your training).

All items on a scale

1 = “no gain”

2 = “a little gain”

3 = “moderate gain”

4 = “good gain”

5 = “great gain”
