# Supplementary material for: Summer Research Internship Curriculum to Promote Self-Efficacy, Researcher Identity, and Peer-to-Peer Learning: Retrospective Cohort Study
Source: JMIR Form Res. 2025 Feb 3;9:e54167. doi: 10.2196/54167 (PMC11809269; doi:10.2196/54167)
Supplement: Multimedia Appendix 4 [file formative-v9-e54167-s004.docx]

Multimedia Appendix 4. Mean Scores Per Respondent for Before Questions Compared to After Questions

| Survey Respondent | Mean Score for *Before* Questions | Mean Score for *After* Questions |
| --- | --- | --- |
| 1 | 3.6 | 4.5 |
| 2 | 2.9 | 4.0 |
| 3 | 2.8 | 4.3 |
| 4 | 2.8 | 3.8 |
| 5 | 3.5 | 4.6 |
| 6 | 1.0 | 4.9 |
| 7 | 3.1 | 4.8 |
| 8 | 1.8 | 3.4 |
| 9 | 3.7 | 4.5 |
| 10 | 2.5 | 5.0 |
| 11 | 4.7 | 5.0 |
| 12 | 2.3 | 4.1 |
| 13 | 3.2 | 3.8 |
| 14 | 2.9 | 4.0 |
